# Environmental species from theTelluria group as the putative origin of bifunctionalβ-lactamases

**DOI:** 10.1128/spectrum.03428-25

**Published:** 2026-07-10

**Authors:** Pedro Penzotti, Gabriel Gutkind, Rachel A. Powers, Pablo Power

**Affiliations:** 1 Facultad de Farmacia y Bioquímica, Universidad de Buenos Aires, Instituto de Investigaciones en Bacteriología y Virología Molecular (IBaViM) 62963 , Buenos Aires, Argentina; 2 Consejo Nacional de Investigaciones Científicas y Técnicas (CONICET) 62873, Buenos Aires, Argentina; 3 Department of Chemistry, Grand Valley State University 1142, Allendale, Michigan, USA; Tel Aviv Sourasky Medical Center, Tel Aviv, Israel

**Keywords:** beta-lactamase evolution, class C beta-lactamases, class D beta-lactamases, LRA-13, *Duganella*, metagenomics

## Abstract

β-Lactamases comprise two structurally and evolutionarily well-defined groups: serine- (SBL) and metallo-β-lactamases (MBL). To date, clinically relevant β-lactamases are typically monofunctional DD-peptidases, containing a single active site cavity per molecule. Recently, several genes encoding putative β-lactamases from the four molecular classes (named as LRA) were identified through functional metagenomics in Alaskan soil samples. *bla*_LRA-13_ encoded a 609-amino acid protein encompassing a class D and a class C-like β-lactamase fused as a single polypeptide, translated from a single open reading frame (ORF). Furthermore, we identified 20 LRA-13 homologs, one of them found in a *Duganella hordei* isolate, sharing 91.3% amino acid identity. Predicted structures generated with AlphaFold 3 showed similar conserved architectures encompassing an N-terminal and C-terminal domains compatible with class D and class C β-lactamases, respectively, connected by a short peptide as a linker and containing their characteristic structural features. A maximum likelihood (ML) evolutionary tree showed a close relationship between LRA-13 and the putative β-lactamase from *Duganella hordei*, a species belonging to the Telluria group, indicating that bifunctional enzymes likely evolved from a common remote ancestor and that their diversification may provide an evolutionary advantage in certain environmental niches. The genetic content of *bla*_LRA-13_ and related genes appears to have a conserved synteny. The description of β-lactamases with two catalytic sites constitutes a novel finding and provides a basis for exploring new evolutionary mechanisms.

**IMPORTANCE:**

β-Lactamases are enzymes able to destroy β-lactam antibiotics and are divided into two main groups according to their structural and mechanistic features: serine- (SBL) and metallo-β-lactamases (MBL). To date, β-lactamases that represent a threat and are produced by bacterial pathogens contain a unique catalytic “pocket,”i.e., only a single β-lactam molecule is bound and cleaved at a time. LRA-13 and other related proteins seem to contain two different catalytic sites of different kinds (one of them is related to class C β-lactamases and the other to class D enzymes). In this study, we analyzed if these enzymes can represent a different evolutionary path for the β-lactamases.

## OBSERVATION

Antimicrobial resistance poses a serious threat to global public health and stands among the most pressing challenges of the 21st^st^ century (1, 2). To date, β-lactamases represent the most important mechanism of resistance to β-lactam antibiotics (3). This enzyme family is highly diverse, comprising more than 11,000 different protein variants according to the β-Lactamase Database (4).

Serine-β-lactamases (SBLs) differentiate from metallo-β-lactamases (MBLs), based on whether their active sites contain a catalytic serine residue or up to two zinc ions. Molecular classification further subdivides SBLs into classes A, C, and D and assigns class B to MBLs, relying on differences in molecular weight and sequence length, active-site architecture, and conserved amino acid motifs within the protein structure (5–7). An important evolutionary pathway that contributes to the emergence of novel β-lactamase variants involves the recruitment of pre-existing genes harbored by environmental microorganisms that inhabit different environments influenced by anthropogenic activity (8). These genes can be mobilized and disseminated to bacterial species more closely associated with clinical settings (9). A well-documented example is the case of CTX-M-type β-lactamases, whose origin has been traced to environmental *Kluyvera* species (10, 11).

Under the One-Health approach, the scientific community extended the study and analysis of antibiotic resistance to environments beyond the clinical setting. In this context, it was found that soil is one of the richest reservoirs of antimicrobial resistance genes (12).

In a notable study by Allen et al., several putative β-lactamase–encoding genes were identified through functional metagenomics in soil samples collected from an island in the Tanana River, Alaska (13). These enzymes, named “LRA” (for “β-**L**actam **R**esistance from **A**laska”), encompassed representatives of all four molecular classes, with a predominance of metallo-β-lactamases (MBLs). Among them, LRA-12 was fully characterized by our group several years ago (14). Remarkably, another of the genes identified, *bla*_LRA-13_, encoded a 609-amino acid protein encompassing a class D and a class C like β-lactamase fused as a single polypeptide translated from a single open reading frame (ORF) and represents the first reported case of a hypothetical bifunctional β-lactamase. Specifically, the genes coding for both the N-terminal (253 amino acids) and C-terminal regions (356 amino acids), which align with class D and C β-lactamases, respectively, as well as the full-length ORF, were subcloned and tested for antibiotic resistance by minimum inhibitory concentration (MIC) determination. All constructs conferred a resistance phenotype to at least one β-lactam antibiotic in recombinant *E. coli* clones: the N-terminal domain (class D) conferred resistance almost exclusively to penicillins like ampicillin and carbenicillin, and the class C domain conferred resistance to cephalosporins like cephalexin. When compared to the β-lactamase sequences available at the time of publication, each module matched class C and D enzymes, with amino acid identity values ranging from 54% to 56%, respectively. However, no significant similarity was found for the full-length ORF (13).

In this work, we extended the search for bifunctional β-lactamases in public databases, performing comparative analyses of their sequences and structural features to reconstruct their evolutionary history in an effort to assess if the occurrence of fusion proteins could represent a potential novel mechanism driving the evolution of these enzymes.

**Fig 1:**
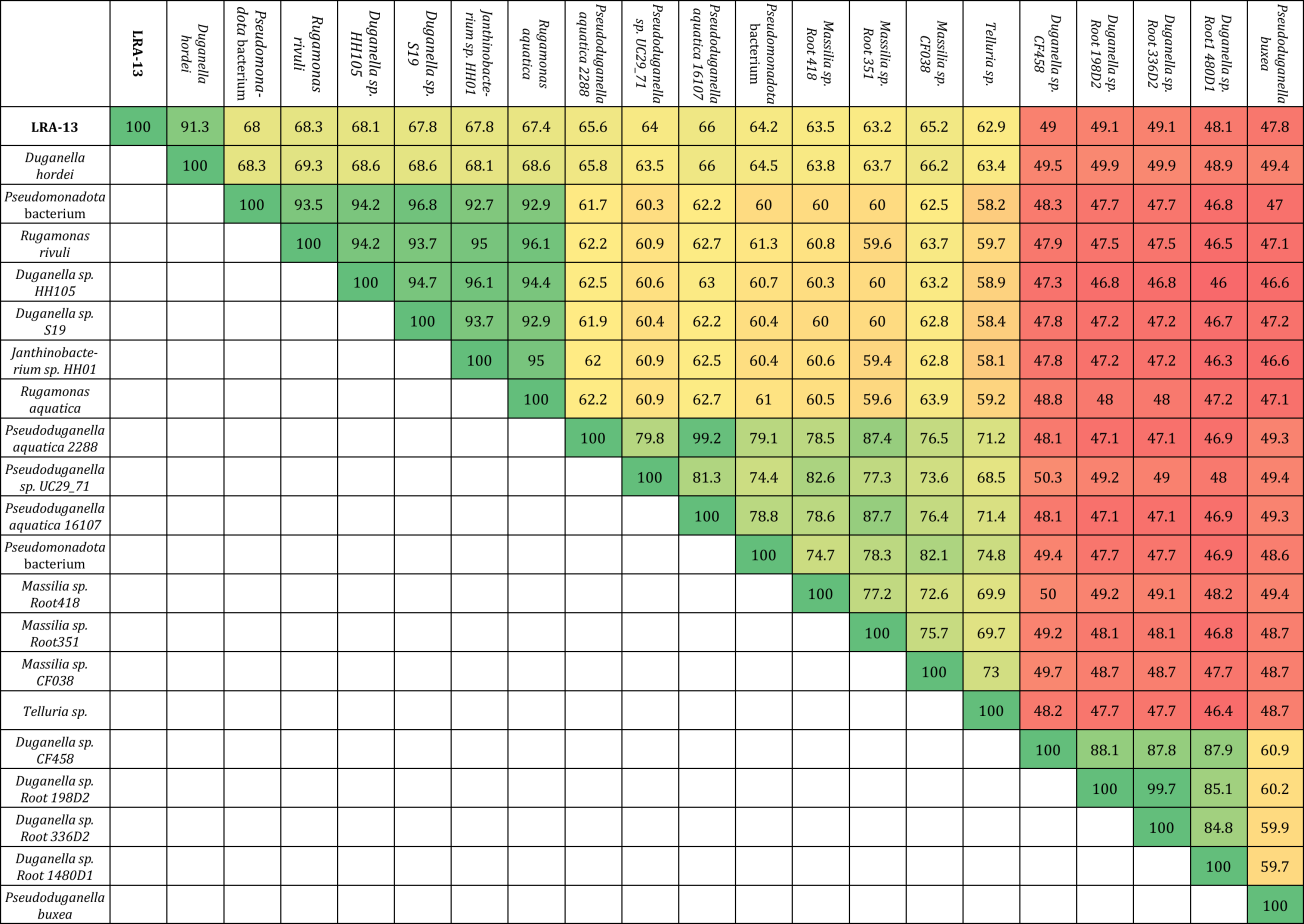
Pairwise amino acid identity (%) among LRA-13 and its closest homologs identified by protein BLAST analysis. Values are represented in a color gradient, with red tones representing values under 50% identity, yellows between 51% and 65% identity, and greens above 66% identity.

By protein BLAST analysis (blastp), we identified 20 homologs with sequence lengths ranging from 597 to 634 amino acids. The most notable hit corresponded to a 609-residue protein from a *Duganella hordei* isolate (accession number:WP_353403837.1) displaying 100% query coverage and 91.3% amino acid identity (Table 1;Fig. 1). The remaining sequences showed 47%–68% amino acid identity and query coverages of 96%–100%.

**TABLE 1:** BLAST matches for LRA-13: query coverage, identity, and accession details

Name	Query coverage	% Identity	Accession length	Accession numbers
*Duganella hordei*	100%	91.30%	609	WP_353403837.1
*Pseudomonadota bacterium*	100%	67.96%	619	MES2161040.1
*Rugamonas rivuli*	100%	68.28%	619	MQA18934.1
*Duganella* sp. HH105	100%	68.12%	619	OEZ55387.1
*Duganella* sp. S19	100%	67.80%	619	WP_332856329.1
*Janthinobacterium* sp. HH01	100%	67.80%	619	WP_008451281.1
*Rugamonas aquatica*	100%	67.42%	619	MQA42609.1
*Pseudoduganella aquatica* 2288	96%	65.59%	620	WP_228895790.1
*Pseudoduganella* sp. UC29_71	99%	63.97%	634	WP_377704066.1
*Pseudoduganella aquatica*16107	96%	66.04%	620	WP_161075628.1
*Pseudomonadota* bacterium	100%	64.20%	614	MES2018019.1
*Massilia* sp. Root418	99%	63.52%	621	KQW93884.1
*Massilia* sp. Root351	100%	63.22%	622	WP_162249946.1
*Massilia* sp. CF038	96%	65.18%	614	WP_083598959.1
*Telluria* sp.	96%	62.86%	618	HEY0065300.1
*Duganella* sp. CF458	96%	48.98%	597	WP_090440397.1
*Duganella* sp. Root198D2	96%	49.07%	597	WP_082591432.1
*Duganella* sp. Root336D2	96%	49.07%	597	WP_082507116.1
*Duganella* sp. Root1480D1	96%	48.05%	597	WP_200951569.1
*Pseudoduganella buxea*	98%	47.84%	623	WP_155472110.1

**Fig 2:**
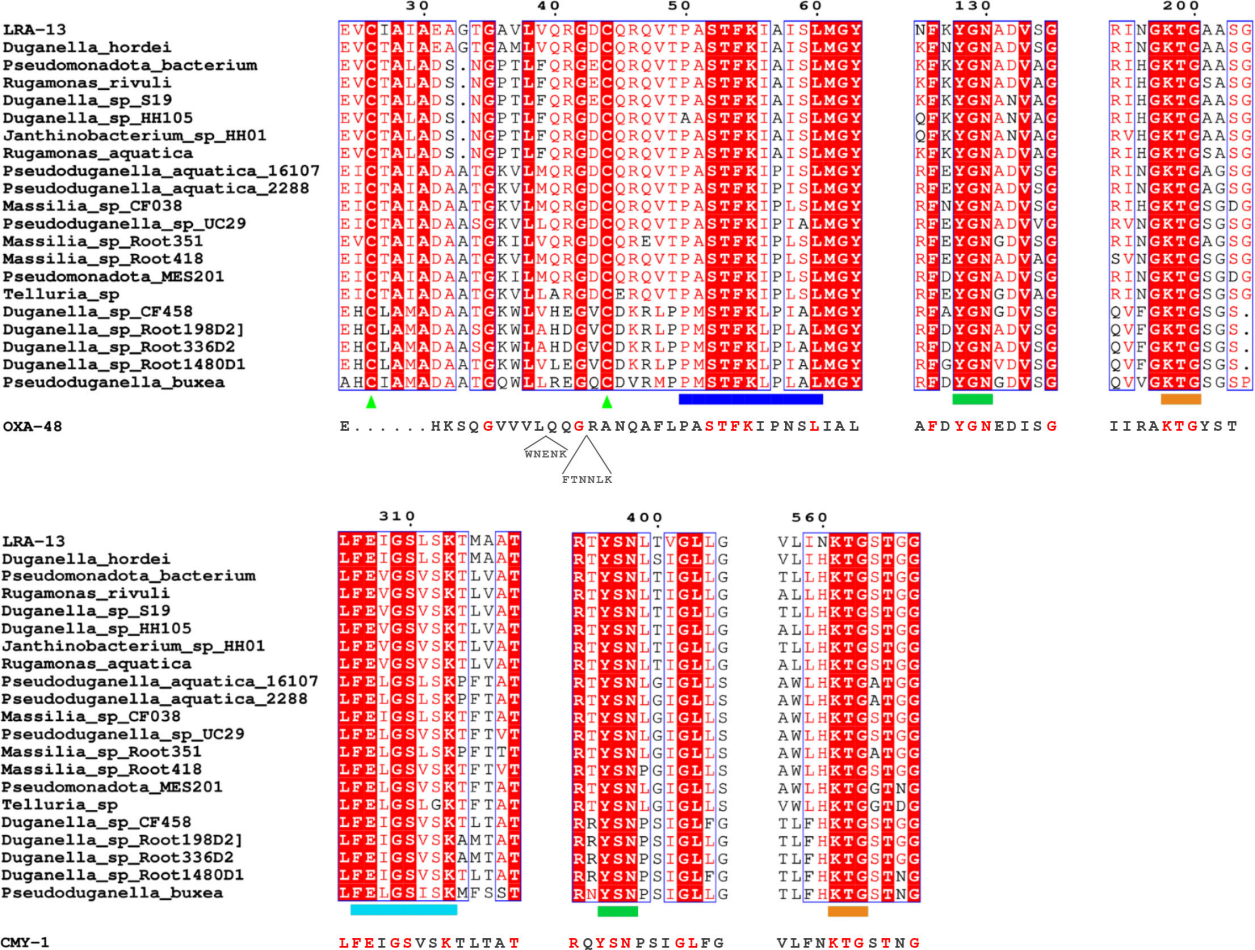
Multiple sequence alignment of key regions in the 21 putative bifunctional β-lactamases. The alignment highlights the most relevant conserved motifs within the class D-like (top) and class C-like (bottom) domains. In the class D-like regions, the PROSITE pattern PS00337 harboring the catalytic serine is shown in blue, the YGN motif in green, and the KTG motif in orange; conserved cysteine residues are indicated with green triangles. In class C-like regions, the PROSITE pattern PS00336 containing the catalytic serine is shown in light blue, the YSN motif in green, and the KTG motif in orange. For reference, the partial sequences of the β-lactamases OXA-48 (class D) and CMY-1 (class C) are shown below the multi-alignment, where matching residues are in red letters; additional residues present in OXA-48 are shown between triangular brackets.

Multiple protein sequence alignment revealed a conserved architecture in all 20 homologs identified: an N-terminal domain compatible with class D β-lactamases and a C-terminal domain compatible with class C β-lactamases, connected by a short peptide that seems to act as a linker. Moreover, inspection of the whole alignment against PROSITE consensus patterns showed that each protein contains two catalytic serine residues, one corresponding to class D (PS00337: [PA]^50^-x-**S**-[ST]-F-K-[LIV]-[PALV]-x-[STA]-[LI]^60^, first and last residues in LRA-13 sequences indicated in superscript numbers) and the other to class C β-lactamases (PS00336: [FY]^308^-E-[LIVM]-G-**S**-[LIVMG]-[SA]-K^315^) (15). The alignment was further examined to verify the presence of the additional conserved motifs shaping the active sites. In class D-like domains, the characteristic triad Y-G-N/S was strictly conserved as Y^128^G^129^N^130^, a motif known to stabilize the transition state during β-lactam hydrolysis, together with the K^198^T^199^G^200^ motif that contributes to the correct orientation of the catalytic serine within the active site. In the class C-like domains, both the K^561^T^562^G^563^ and the Y-X-N motifs were conserved, the latter specifically conserved as Y^396^S^397^N^398^ (Fig. 2) (16).

The only two cysteine residues in the LRA-13 sequence align with conserved cysteine positions across the 21 β-lactamases examined (Fig. 2), suggesting that a disulfide bond may contribute to the proper folding and stability of these enzymes. In addition, SignalP 5.0 analysis of all 21 proteins predicted a Sec/SPI signal peptide, a standard secretory peptide routed through the Sec translocon and cleaved by Signal Peptidase I. This prediction is consistent with the periplasmic localization typically observed for β-lactamases in gram negatives.

**Fig 3:**
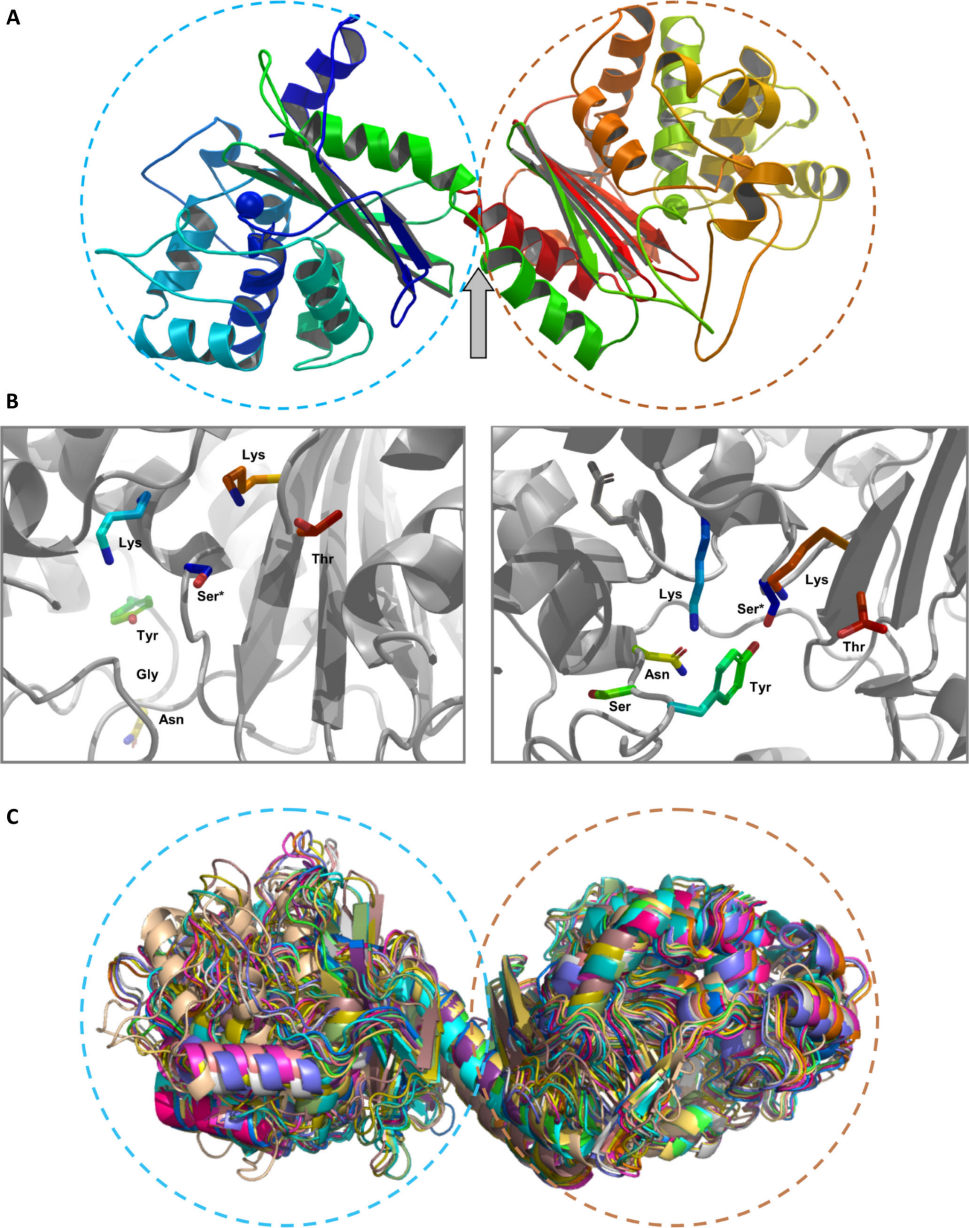
AlphaFold-predicted structures of LRA-13 and its 20 putative bifunctional β-lactamase homologs. (**A**)Structural model of LRA-13 predicted with AlphaFold. The N-terminal class D–like domain (light blue circle) and the C-terminal class C–like domain (brown circle) are connected by a linker peptide (gray arrow). Catalytic serine residues located within the active sites of both domains are highlighted as spheres. (**B**)Active-site architecture of the class D–like domain (left) and the class C–like domain (right). The catalytic serine (Ser*) and the lysine residue that define the active site are shown in blue; the tyrosine, asparagine, and serine residues forming the Y-X-N motif are depicted in green and yellow; and the threonine and lysine residues forming the K-T-G motif are indicated in orange and red, respectively. (**C**)Superposition of the 21 AlphaFold-predicted bifunctional β-lactamase models. Both domains maintain the characteristic conserved α/β core architecture. A short linker peptide connecting the two modules is observed in the center of the structure, acting as a bridge between the class D (light-blue circle) and class C domains (brown circle).

In the LRA-13 model (Fig. 3A and B), the class D and class C domains are clearly delineated, separated by an interdomain linker (arrow) that positions each active site for independent substrate binding and hydrolysis. Superposition of all 21 AlphaFold models in PyMOL (Fig. 3C) revealed that, despite amino acid identities dropping to as low as 46% in some cases, both domains maintain a conserved α/β core, indicating structural preservation of the catalytic sites in all of them. Variable loop regions likely reflect substrate-specific adaptations, while the flexible linker region appears essential for the spatial arrangement of the two modules.

To test if LRA-13 and the individual class C and D domains can hydrolyze β-lactam substrates, we determined the specific activity on clear crude extracts. As shown in Table 2, the activity against cephalosporins is clearly granted to the class C domain, while the hydrolysis of ampicillin is mostly due to the class D domain. These results confirm that LRA-13 is a fully active β-lactamase, where each individual domain contributes to preferential hydrolytic activity either to penicillins (class D) or cephalosporins (class C), as was already suggested by Allen et al*.* (13). Further biochemical and structural studies are needed to evaluate the actual contribution of each catalytic site in the interaction with β-lactams.

**TABLE 2:** Hydrolysis rate and specific activity of LRA-13 and individual class C and class D enzymes against reference β-lactam substrates

		LRA-13	Class D domain	Class C domain
Nitrocefin	Specific activity (µmol/mg min)	26.83	55.98	91.72
Cephalothin	Specific activity (µmol/mg min)	2.90	0.14	14.84
Ampicillin	Specific activity (µmol/mg min)	9.66	43.67	0.05

**Fig 4:**
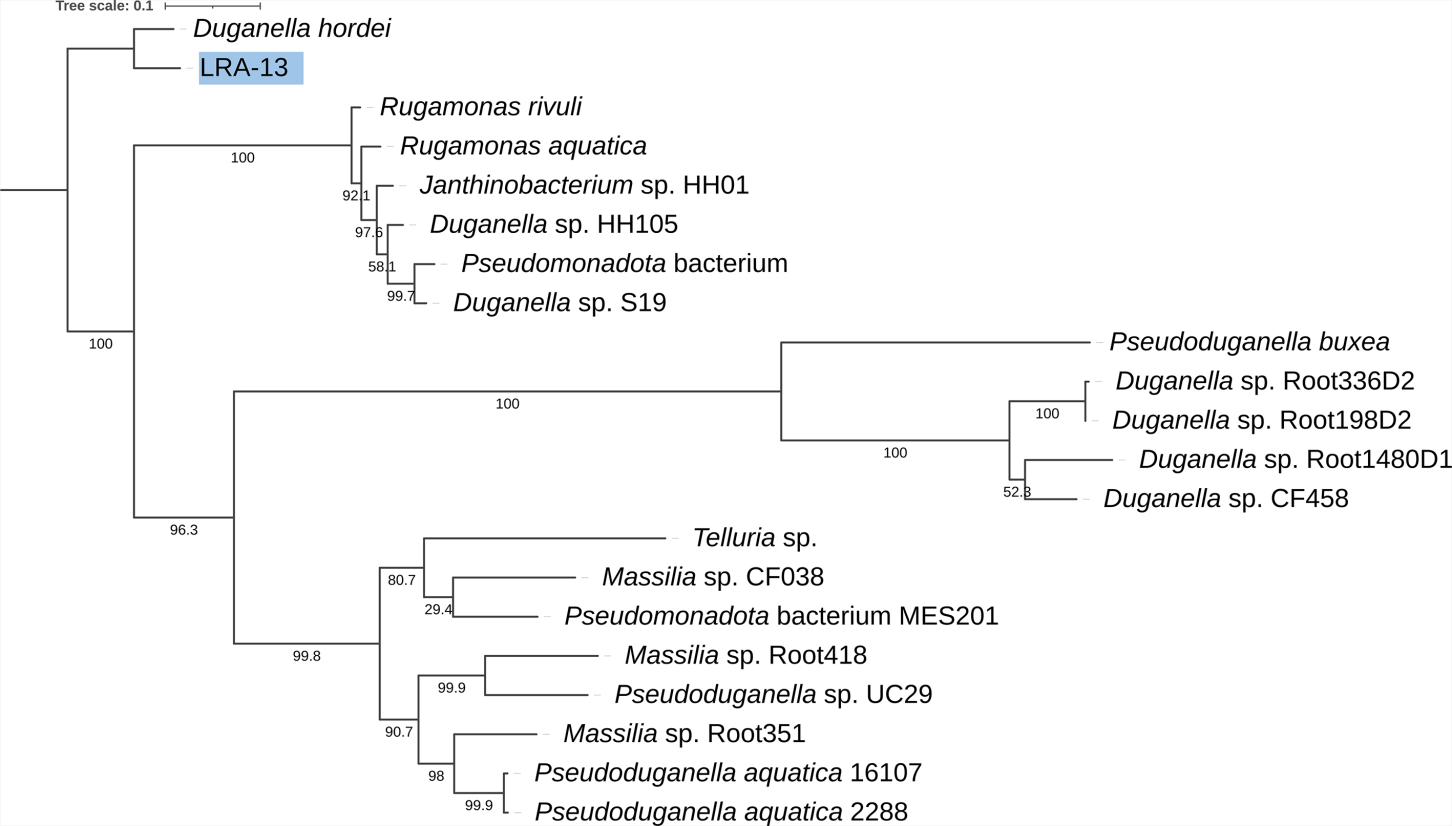
Maximum-likelihood evolutionary tree of bifunctional β-lactamases: At the top of the tree, LRA-13 (highlighted in blue) clusters with the β-lactamase from *Duganella hordei* in a well-supported clade that also includes other *Rugamonas* species and related environmental genera (*Duganella* and *Janthinobacterium*). Additional clades group bifunctional β-lactamases from *Duganella*, *Pseudoduganella*, and *Massilia* spp. The tree was inferred with IQ-TREE under the LG+G4 model. Branch support values are shown as SH-aLRT/UFBoot.

The close relationship between LRA-13 and the putative β-lactamase from *Duganella hordei*, recovered from a plant sample, is clear (Fig. 4). This phylogenetic proximity is in agreement with the high amino acid identity between the two enzymes. Both sequences cluster in a well-defined clade at the top of the tree, with variants from other *Rugamonas* species and environmental genera such as *Duganella* spp. and *Janthinobacterium* spp. The remaining clades mainly comprise other *Duganella* members and environmental species like *Pseudoduganella* spp. and *Massilia* spp.; however, the taxonomic boundaries of these groups have been highly dynamic in recent years, as discussed in the following sections.

Overall, these bifunctional enzymes appear to have evolved from a common remote ancestor, and their subsequent diversification, under different selective pressures, suggests that the coexistence of two likely functional active sites within a single protein may provide an evolutionary advantage in the environmental niches occupied by these bacteria.

To trace the evolutionary origin of these bifunctional β-lactamases, we examined the bacterial species where these enzymes were detected: *Duganella* sp., *Pseudoduganella* sp., *Janthinobacterium* sp., *Rugamonas* sp., *Telluria* sp., and *Massilia* sp. Interestingly, all belong to the family *Oxalobacteraceae* (order Burkholderiales, subclass Betaproteobacteria) and are members of the Telluria group (NCBI Taxonomy ID: 2895353) within this family (17). We also noted that taxonomic classification within *Oxalobacteraceae* has been highly dynamic in recent years: for example, an entire genus (*Rugamonas* spp.) was recently incorporated into the family, and many species formerly assigned to *Pseudoduganella* sp. have since been reclassified under *Massilia* sp. (18–21).

**Fig 5:**
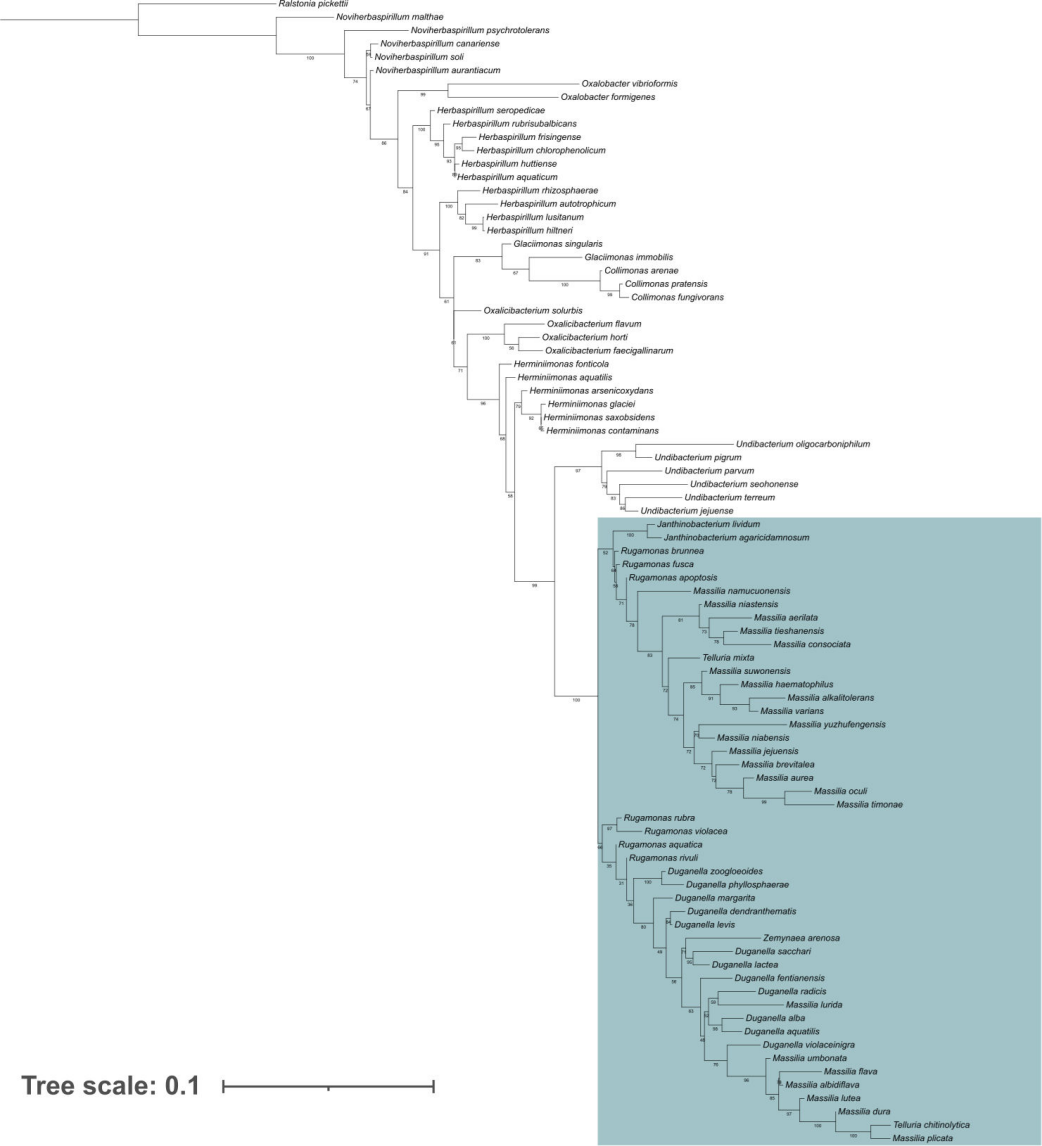
Maximum-likelihood phylogenetic tree of the *Oxalobacteraceae* family: The phylogenetic tree was constructed from representative 16S rRNA gene sequences of type strains from the family *Oxalobacteraceae*. Species harboring the analyzed bifunctional β-lactamases cluster within the Telluria group (shaded in light blue). The tree was inferred by using IQ-TREE under the K3Pu+F + G4 model, with *Ralstonia pickettii* as the outgroup. Branch support values are shown as SH-aLRT/UFBoot.

The resulting tree based on 16S rRNA gene sequences from *Oxalobacteraceae* representatives (Fig. 5) highlights how the microorganisms that harbor the bifunctional β-lactamase cluster within the Telluria group (shaded in light blue). This supports the hypothesis that this group represents the evolutionary origin of these novel enzymes.

**Fig 6:**
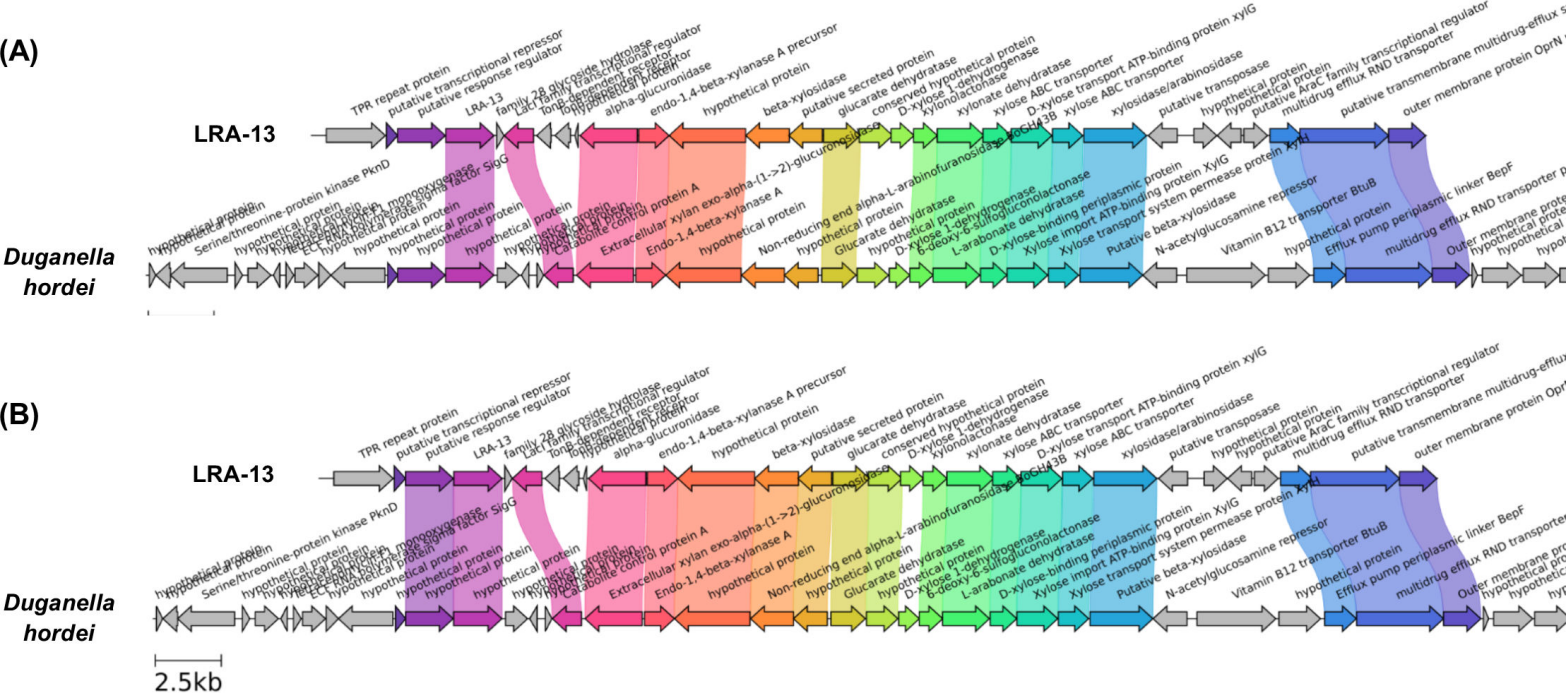
Genomic context comparison of the bifunctional β-lactamase genes. Comparison between the contig carrying *bla*_LRA-13_ and the one recovered from *Duganella hordei* at a clustering threshold of 0.90 (**A**)and 0.85 (**B**)Colored blocks indicate homologous gene clusters identified by Clinker.

Finally, we analyzed the genetic contexts of *bla*_LRA-13_ and homologous genes. When applying a 0.85 identity threshold to assign genes to the same cluster (Fig. 6A), we observed a marked conservation of the genomic contexts, and synteny was preserved even when the threshold was increased to 0.90 (Fig. 6B). Table 3 lists, in order of appearance, the genes constituting the genetic context of the bifunctional β-lactamases. Most of the genes located downstream are associated with transport and metabolism of various compounds. Notably, a putative response regulator is present upstream of the bifunctional β-lactamases, which may be involved in their activation or repression in response to certain signals, such as the presence of β-lactam antibiotics.

**TABLE 3:** Functionally annotated genes shared between the two contigs at the 0.85 similarity threshold^*a*^

Functionally annotated protein	Predicted function	Threshold	
		0.90	0.85
Putative response regulator	Transcriptional regulator	−	+
LRA-13	Bifunctional β-lactamase	+	+
LacI family transcriptional regulator	Transcriptional regulator	+	+
Alpha-glucuronidase	Metabolism	+	+
Endo-1,4-beta-xylanase A precursor	Transport and metabolism	+	+
Hypothetical protein	Unknown	+	+
Beta-xylosidase	Metabolism	−	+
Putative secreted protein	Unknown	−	+
Glucarate dehydratase	Metabolism	+	+
Conserved hypothetical protein	Unknown	−	+
Xylonolactonase	Metabolism	+	+
Xylonate dehydratase	Metabolism	+	+
Xylose ABC transporter	Transport	+	+
D-xylose transport ATP-binding protein XylG	Transport	+	+
Xylose ABC transporter	Transport	+	+
Xylosidase/arabinosidase	Metabolism	+	+
Multidrug efflux RND transporter	Transport	+	+
Putative transmembrane multidrug- efflux system lipoprotein transmembrane	Transport	+	+
Outer membrane protein OprN precursor	Transport	+	+

^*a*^ Genes are listed in order of appearance, with the annotated protein name, predicted function, and presence (+)/absence (−) at the 0.85 and 0.90 thresholds indicated.

Our findings demonstrate that, beyond the first report of a bifunctional β-lactamase recovered from a functional metagenome, multiple enzymes with similar features are present in public databases. All share a conserved modular organization: two catalytic domains, class D at the N-terminus and class C at the C-terminus, separated by a likely flexible interdomain linker. Each domain contains not only the PROSITE-defined patterns used to classify them as class D and class C β-lactamases but also the conserved motifs that define the characteristic active site of these enzymes. Our study expands on previous findings reported by Silveira et al. (22)

Moreover, structural superposition of AlphaFold models indicates that this fold is preserved even when amino acid sequence identity between pairs is as low as ~50%. The restriction of these bifunctional enzymes to species within the Telluria group of the *Oxalobacteraceae* family suggests that this clade represents their evolutionary origin. Comparative analysis of the genetic contexts flanking the LRA-13 gene and its counterpart in *Duganella hordei* further reveals conserved synteny, even at stringent similarity thresholds, supporting the existence of a relatively recent common ancestor.

So far, it was generally accepted that β-lactamases have evolved from the penicillin-binding proteins (PBPs), many of which are bifunctional enzymes (i.e., trans-glycosylase and DD-transpeptidase) (23). In fact, clinically relevant β-lactamases are typically monofunctional DD-peptidase enzymes as they contain a single active site cavity per protein molecule (6). Therefore, the description of β-lactamases with two catalytic sites not only constitutes a novel finding in this field but could also provide a basis for exploring new evolutionary mechanisms, where the emergence of an enzyme with two catalytic sites may result from the fusion of two ancestral monofunctional enzymes or, conversely, an enzyme with two independent modules may, through different molecular mechanisms, give rise to two separate proteins.

Future studies focusing on functional characterization and environmental distribution will be essential to elucidate the ecological and evolutionary pressures that gave rise to this unique enzymatic innovation.

Additionally, a revision and systematization of the nomenclature of the LRA β-lactamases could be useful to assign them to pre-existing or new families of the four molecular classes.

### Sequence analysis

A protein BLAST (BlastP) search was performed using the full-length 609-amino-acid sequence of LRA-13 (accession number:WP_063839877.1) as input, against the NCBI’s nonredundant protein sequences database (ClusteredNR) and the default parameters (BLOSUM62 matrix, gap costs 11/1, and conditional compositional score matrix adjustment) (24). Only those sequences that covered both Class D and Class C domains were selected for subsequent analyses.

A multiple protein sequence alignment was performed with MAFFT v7.520 using the L-INS-i iterative refinement algorithm and the BLOSUM62 substitution matrix, with default parameters applied for all other settings (25). For visualization and highlighting of conserved regions and motifs, the alignment was processed with ESPript v3.0 (26).

Prediction of signal peptides and cellular localization was performed with SignalP v5.0, using the full amino acid sequence of each protein as input (27).

Comparative analysis of the genetic contexts of both *bla*_LRA-13_ and the homologous gene from the *Duganella hordei* isolate was achieved by the Clinker tool based on the prior annotation of the genes contained in the corresponding contigs (28).

### *In silico* structural modeling

Predicted structures of all 21 putative β-lactamases were generated with AlphaFold3 using default parameters in order to evaluate domain organization and conserved motifs. Model quality was assessed using pLDDT scores (29). These structures were visualized and superimposed using PyMOL version 3.1.6.1 (30).

### Phylogeny

Based on the set of aligned full-length amino acid sequences, a maximum-likelihood (ML) evolutionary tree was inferred using IQ-TREE v2.2.2.7 under the LG+G4 model selected by ModelFinder (BIC) (31). Also, a maximum-likelihood (ML) phylogenetic tree based on 16S rRNA gene sequences from *Oxalobacteraceae* representatives was inferred using IQ-TREE v2.2.2.7 under the K3Pu+F + G4 model selected by ModelFinder; *Ralstonia pickettii* was selected as an outgroup species. In both cases, branch support was assessed with 1,000 ultrafast bootstrap replicates (UFBoot) and 1,000 SH-aLRT replicates, applying thresholds of UFBoot ≥ 70% and SH-aLRT ≥ 80 %.

### β-Lactamase expression and determination of enzyme activity

Recombinant plasmids encoding LRA-13 in its complete sequence and individual class C and class D modules, using the backbone and expression system of inducible pET28a(+) vector (kanamycin resistance), were obtained by gene synthesis (Gene Universal Inc, Newark, DE, USA). The synthetic plasmids were introduced into*E. coli* BL21(DE3) cells by chemical transformation, and overnight cultures were prepared in 100 mLLysogeny broth (LB) supplemented with 30 µg/mL kanamycin and 1 mM IPTG to induce β-lactamase expression. Crude extracts containing the β-lactamases were obtained through sonication and further centrifugation (10,000 × *g* for 20 min) for eliminating cell debris. The whole protein concentration in clear crude extracts was assessed by measuring the absorbance at 280/260 nm and using the Warburg-Christian equation: protein concentration (mg/mL) = 1.55 × Abs_280nm_ - 0.76 × Abs_260nm_. β-Lactamase activity was measured by spectrophotometric monitoring of antibiotic hydrolysis in a Shimadzu UV-1900i at 25°C. The concentration and wavelengths used for each antibiotic were as follows: 80 µM cephalothin at 273 nm (Δε = −6,300 M^−1^ cm^−1^); 350 µM ampicillin at 235 nm (Δε = −820 M^−1^ cm^−1^); 100 µM nitrocefin at 482 nm (Δε = +15,000 M^−1^ cm^−1^). The hydrolysis rate was determined by the linear regression plot of the absorbance variation at the corresponding wavelength during the first 30 s hydrolysis, using the Lambert-Beer equation. One unit of β-lactamase activity was defined as the amount of enzyme which hydrolyzes 1 µmol of substrate per min at 25°C, and the specific activity was defined as the units of β-lactamase per milligram of protein.
